# Protective effects of *Dunaliella salina* – a carotenoids-rich alga – against ultraviolet B-induced corneal oxidative damage in mice

**Published:** 2012-06-13

**Authors:** Chia-Fang Tsai, Fung-Jou Lu, Yu-Wen Hsu

**Affiliations:** 1 Department of Biotechnology, TransWorld University, Douliu City, Taiwan, ROC; 2 Institute of Medicine, College of Medicine, Chung Shan Medical University, Taichung City, Taiwan, ROC; 3 School of Optometry, Chung Shan Medical University, Taichung City, Taiwan, ROC; 4 Department of Ophthalmology, Chung Shan Medical University Hospital, Taichung City, Taiwan, ROC

## Abstract

**Purpose:**

Ultraviolet B (UVB) radiation from sunlight is known to be a risk factor for human corneal damage. The purpose of this study was to investigate the protective effects of *Dunaliella salina* (*D. salina*) on UVB radiation-induced corneal oxidative damage in male imprinting control region (ICR) mice.

**Methods:**

Corneal oxidative damage was induced by exposure to UVB radiation at 560 μW/cm^2^. Animals were orally administered (gavage) *D. salina* at doses of 0, 123, and 615 mg/kg bodyweight/day for eight days. Corneal surface damages were graded according to smoothness and the extent of lissamine green staining. Corneal glutathione (GSH) and malondialdehyde (MDA) levels, as well as the activities of superoxide dismutase (SOD), catalase, glutathione peroxidase (GSH-Px), and glutathione reductase (GSH-Rd) in cornea were measured to monitor corneal injury.

**Results:**

UVB irradiation caused significant damage to the corneas, including apparent corneal ulcer and severe epithelial exfoliation, leading to decrease in the activities of SOD, catalase, GSH-Px, GSH-Rd, and GSH content in cornea, whereas there was increased corneal MDA content as compared with the control group. Treatment with *D. salina* could significantly (p<0.05) ameliorate corneal damage and increase the activities of SOD, catalase, GSH-Px, GSH-Rd, and GSH content, and decrease the MDA content in corneas when compared with the UVB-treated group.

**Conclusions:**

The studies demonstrate that *D. salina* exhibits potent protective effects on UVB radiation-induced corneal oxidative damage in mice, likely due to both the increase of antioxidant enzyme activity and the inhibition of lipid peroxidation.

## Introduction

Ultraviolet (UV) irradiation is the most common cause of radiation injury to the eye. The cornea has the physiologic capacity to absorb the majority of UVB radiation, and protects the inner eye against UVB-induced oxidative damaging effects. A recent study has suggested that the cornea absorbs 92% of UVB and 60% of UVA radiation and is most sensitive to UVB damage [1]. The corneal effects of excessive exposure to UVB radiation may include photokeratitis, damage to the epithelium, edema, and several biochemical changes, including DNA modification, protein cross-linking, enzyme inactivation, and the production of excessive reactive oxygen species (ROS) [2-4]. Previous reports suggested that natural antioxidants can effectively prevent and cure UVB-induced cell damage in cornea [5]. Antioxidants appear to act against oxidative stress by raising the levels of endogenous defense (e.g., by upregulating gene expressions of the antioxidant enzymes, such as superoxide dismutase (SOD), catalase and glutathione peroxidase [6].

*Dunaliella salina* (*D. salina*) is a unicellular biflagellate green alga of the Chlorophyceae class. The algal cells are surrounded by a thin elastic membrane and can yield three major valuable products: glycerol, β-carotene, and proteins [7]. Due to the abundance of β-carotene, which is an antioxidant as well as a vitamin A precursor, *D. salina* has been used as a food coloring agent, a pro-vitamin A food supplement, an additive to food and cosmetics, and a health food product [8-12]. Recently, our group demonstrated that the major carotenoids in *D. salina* include all-*trans*-β-carotene and 9- or 9’-*cis*-β-carotene. Specifically, the 9-cis isomer has demonstrated a higher antioxidant activity due to the higher reactivity of the cis bond compared to trans. By trolox equivalent antioxidant capacity assay, reducing power, and 2, 2-diphenyl-2-picrylhydrazyl hydrate radical scavenging assay, we also found that our carotenoid-rich algal extract had significantly higher antioxidant activity than pure all-trans-β-carotene, α-carotene, lutein, and zeaxanthin [13]. Both lutein and zeaxanthin are major constituents of the retinal macular region of humans [14]. Increased dietary intake of lutein and zeaxanthin was found to result in increased plasma levels, which were positively associated with a reduced risk for age-related macular degeneration [15]. Additionally, the major precursor of vitamin A is β-carotene, which quenches excited sensitizer molecules and singlet oxygen. Documented evidence has been reported that vitamin A deficiency is known to cause a high degree of damage in ocular surfaces [16].

Based on the excellent antioxidant activities of *D. salina* found in vitro, it was of interest to us to evaluate its protective effects in vivo. In the present study, male ICR mice were orally treated with *D. salina* daily, accompanied by UVB exposure for a period of eight days. Corneal surface damage was graded according to corneal smoothness and the extent of lissamine green staining. Corneal glutathione (GSH) and malondialdehyde (MDA) levels, as well as SOD, catalase, glutathione peroxidase (GSH-Px), and glutathione reductase (GSH-Rd) activities in cornea tissues, were also measured to monitor corneal injury.

## Methods

### *D. salina* material

Commercially available spray-dried preparations of *D. salina* cultured in the outdoor cultivation pool at GONG BIH Enterprise Co., Ltd. (Yunlin City, Taiwan, ROC) were suspended in distilled water before use. The quality of *D. salina* powder was described and provided by the company. The carotenoid contents in the *D. salina* were measured as described previously [13].

### Animals

Male imprinting control region (ICR) mice (22±2 g; 5 weeks old) were obtained from the Animal Department of BioLASCO Taiwan Co., Ltd. (Taipei City, Taiwan, ROC). Animals were quarantined and allowed to acclimate for one week before beginning experimentation. Animals were housed 3–4 per cage under standard laboratory conditions with a 12 h light/dark cycle. The animal room temperature was maintained at 25±2 °C with a relative humidity of 55±5%. Air handling units in the animal rooms were set to provide approximately 12 fresh air changes per hour. A standard rodent diet (Rodent LabDiet 5001; PMI Nutrition International, LLC, Richmond, IN) was used for these studies. Appropriate analyses for the constituents and nutrients were performed by the manufacturer and provided to the Laboratory Animal Center, Chung Shan Medical University (Taichung City, Taiwan, ROC). Food and water were available ad libitum. The experimental protocols for this study were approved by the Institutional Animal Care and Use Committee, and the animals were cared for in accordance with the institutional ethical guidelines. All procedures were performed according to the ARVO Statement for the Use of Animals in Ophthalmic and Vision Research.

### Treatment

**Figure 1:**
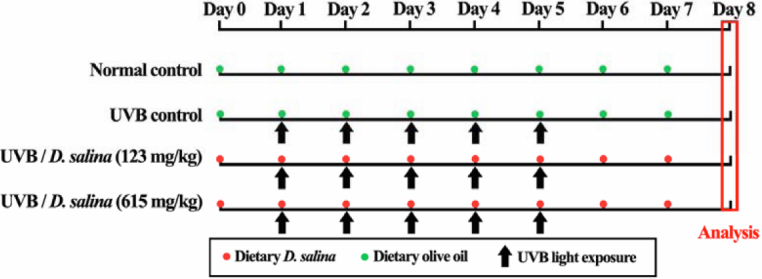
Experimental protocol for dietary *D. salina* supplementation after UVB irradiation to the mouse cornea. Daily UVB light exposure (indicated by arrows) was performed from Day 1 to Day 5, with dietary *D. salina* given at 123 and 615 mg/kg of bodyweight, respectively, from Day 0 to Day 7 (indicated by red point). The normal control group and the UVB control group were given olive oil from Day 0 to Day 7 (indicated by green point).

The animals were randomly divided into four groups, each consisting of 10 mice. Group I served as the normal control and was given olive oil by gavage daily for a period of eight days (Day 0 to Day 7 in Figure 1). To induce corneal damage in vivo, eyes of the animals in Groups II, III, and IV were exposed to UVB irradiation using the method of Tanito and colleagues [17] with slight modification. After anesthesia was induced by intraperitoneal injection of sodium pentobarbital (60 mg/kg bodyweight), both eyes were exposed to 560 μW/cm^2^ of UVB light (UVLS-26; UVP Inc., Cambridge, UK) for 180 s in a darkroom. During irradiation, the mice were confined in an adjustable retaining cage that protected most of the animal, except the head, from the UV light. The wavelength of the light source peaked at 312 nm (range, 280–315 nm). The energy output was measured with a UV detector (USB4000) with a sensor (CC-3-UV-S; both from Ocean Optics, Inc., FL). The entire UVB irradiation course was completed in a consecutive five-day period (Day 1 to Day 5 in Figure 1). Group II served as the UVB control and was exposed to UVB irradiation daily for a period of five days, with daily olive oil treatment for a period of eight days (Day 0 to Day 7 in Figure 1). Groups Ш and IV were exposed to UVB irradiation daily for a period of five days, and were orally administered the *D. salina* dissolved in olive oil at doses of 123 and 615 mg/kg, respectively, daily, for a period of eight days (Day 0 to Day 7 in Figure 1). At the end of the experiment, the animals were anesthetized and evaluated for corneal damage by dissection microscope. After assessment of the corneal damage, all animals were sacrificed by CO_2_ for euthanasia. Eye samples were dissected out and washed immediately with ice-cold saline to remove as much blood as possible, and immediately stored at −70 °C until further analysis.

### Evaluation of corneal damage

Seventy-two hours after UVB exposure, the digitized images of the mouse corneas were obtained with a dissection microscope (SZ-PT; Nikon, Tokyo, Japan) equipped with a digital camera (Coolpix P5000; Nikon). To obtain the image of the cornea, a ring-shaped light source (FC100; Meike, Taichung City, Taiwan, ROC) was attached to the dissection microscope, and the light was projected to the center of the cornea when the images were obtained. To evaluate corneal surface irregularities caused by UVB exposure, mire irregularity, which is thought to reflect corneal surface integrity, was quantified based on the method by Tanito and colleagues [17]. The corneal surface irregularity was graded 0–3 as follows: absent (grade 0), mild (grade 1), moderate (grade 2), and severe (grade 3). After corneal smoothness was scored, either the right or the left eye was randomly selected and stained with 1% lissamine green (Sigma-Aldrich, St. Louis, MO). The digitized images of lissamine green staining on the corneal surface were taken and scored according to the method published by Chen and colleagues [18]. Briefly, the total area of punctuate staining was designated as grade 0; grade 1, less than 25% of cornea stained with scattered punctuate staining; grade 2, 25%–50% of cornea stained with diffuse punctuate staining; grade 3, 50%–75% of cornea stained with punctuate staining and apparent epithelial defects; grade 4, more than 75% of cornea stained with abundant punctuate staining and large epithelial defects. The final numerical score was calculated by dividing the sum of the number per grade of affected mice by the total number of examined mice. All scorings were performed by two observers without prior knowledge of the UVB exposure and study groups.

### Measurement of lipid peroxidation

The quantitative measurement of lipid peroxidation was done by measuring the concentration of thiobarbituric acid reactive substances (TBARS) in cornea using the method of Berton and colleagues [19]. The amount of malondialdehyde (MDA) formed was quantitated by reaction with thiobarbituric acid (TBA) and used as an index of lipid peroxidation. In brief, samples were mixed with a TBA reagent consisting of 0.375% TBA and 15% trichloroacetic acid in 0.25 N hydrochloric acid. The reaction mixtures were placed in a boiling water bath for 30 min and centrifuged at 1,811× g for 5 min. The supernatant was collected and its absorbance was measured at 535 nm. The results were expressed as nmole/mg protein using the molar extinction coefficient of the chromophore (1.56×10^−5^ M^−1^cm^−1^).

### Measurement of SOD, catalase, GSH-Px, GSH-Rd, and GSH in corneal homogenate

Corneal homogenates were prepared in cold Tris-HCl (5 mmol/l, containing 2 mmol/l EDTA, pH 7.4) using a homogenizer. The unbroken cells and cell debris were removed by centrifugation at 11,180× g for 10 min at 4 °C. The supernatant was used immediately for the assays for SOD, catalase, GSH-Px, GSH-Rd, and GSH. The activities of all of these enzymes, and the GSH levels, were determined following the instructions in the Randox Laboratories Ltd. kit (Antrim, United Kingdom).

### Statistical analysis

All values are expressed as the mean±SD. Comparison between any two groups was performed using a χ^2^ or one way ANOVA (ANOVA) followed by Dunnett multiple comparison tests using the statistical software SPSS (DR Marketing Co., Ltd. New Taipei City, Taiwan, ROC). Statistically significant differences between groups were defined as p<0.05.

## Results

### Effect of *D. salina* on UVB-induced corneal damage

**Figure 2:**
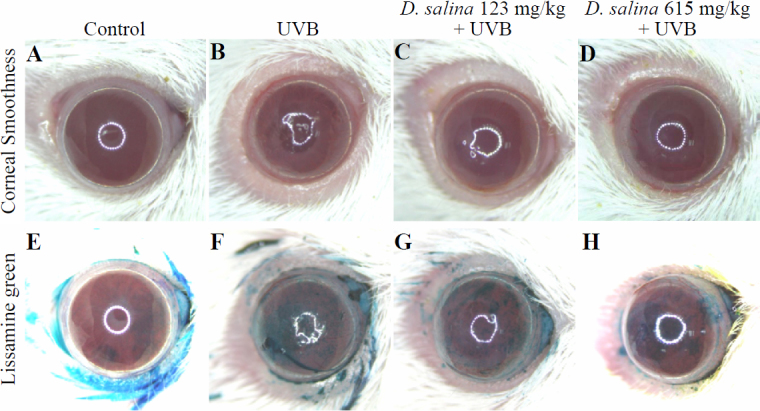
Effects of *D. salina* on UVB radiation-induced corneal damage. Comparison of corneal smoothness and lissamine green staining among control, UVB, and UVB/*D. salina* (UVB exposure with dietary *D. salina* at 123 and 615 mg/kg of bodyweight, respectively) groups.

Cornea surface examination provided direct evidence of corneal damage cause by UVB irradiation. Lissamine green is a routine staining dye for evaluation of corneal damage in clinical diagnosis and experimental examination. The results of cornea surface examination are shown in Figure 2. UVB irradiation caused serious damage on the corneal surface (Figure 2A), including apparent corneal ulcer, severe epithelial exfoliation, and deteriorated corneal smoothness, as compared to normal controls (Figure 2B). In contrast, a significant amelioration was observed in corneal surface examination in the groups treated with *D. salina* (Figure 2C,D) in comparison with those observed in the UVB-treated group. With lissamine green staining, the dark-blue devitalized epithelial areas on the ocular surface were obvious in the eyes from the UVB group (Figure 2F), indicating that UVB induced serious damage to the corneal surface. In contrast, no dark-blue devitalized epithelial areas were found in the eyes from the normal control group (Figure 2E). Compared with the lesions observed in the UVB-treated group, mild and trace degrees of corneal ulcer, epithelial exfoliation, and deteriorated corneal smoothness were observed on the ocular surface of *D. salina*-treated mice at doses of 123 and 615 mg/kg, respectively (Figure 2G,H).

**Figure 3:**
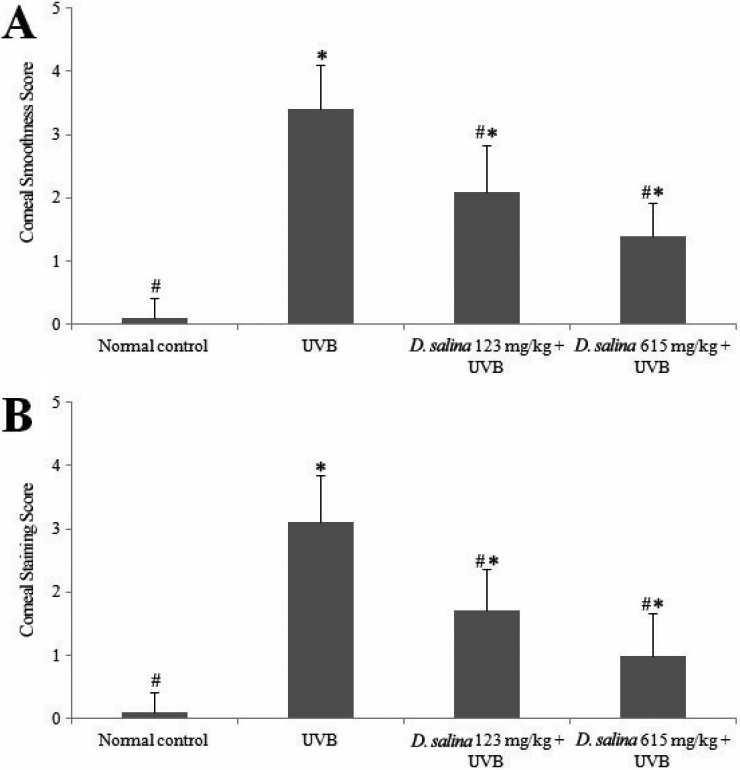
Corneal mire grade and the lissamine green staining index. Effects of *D. salina* on corneal mire grade (**A**) and the lissamine green staining index (**B**) in UVB-induced corneal oxidative damage in mice. * p<0.05 compared with normal control. # p<0.05 compared with UVB-treated alone group. All data are expressed as the mean±SD.

Cornea surface examinations for corneal smoothness and lissamine green staining were recorded and scored, as shown in Figure 3. In this semi-quantitative assessment, all scores of cornea surface examination in the UVB-treated group were significantly higher than those of the normal control (p<0.05), indicating that UVB had induced severe damage to the cornea. All of the tested doses of *D. salina* significantly decreased (p<0.05) the scores of corneal smoothness and lissamine green staining as compared to the UVB-treated group, indicating that *D. salina* ameliorated UVB-induced corneal damage.

### Effect of *D. salina* on MDA and GSH levels after UVB exposure in the cornea

**Figure 4:**
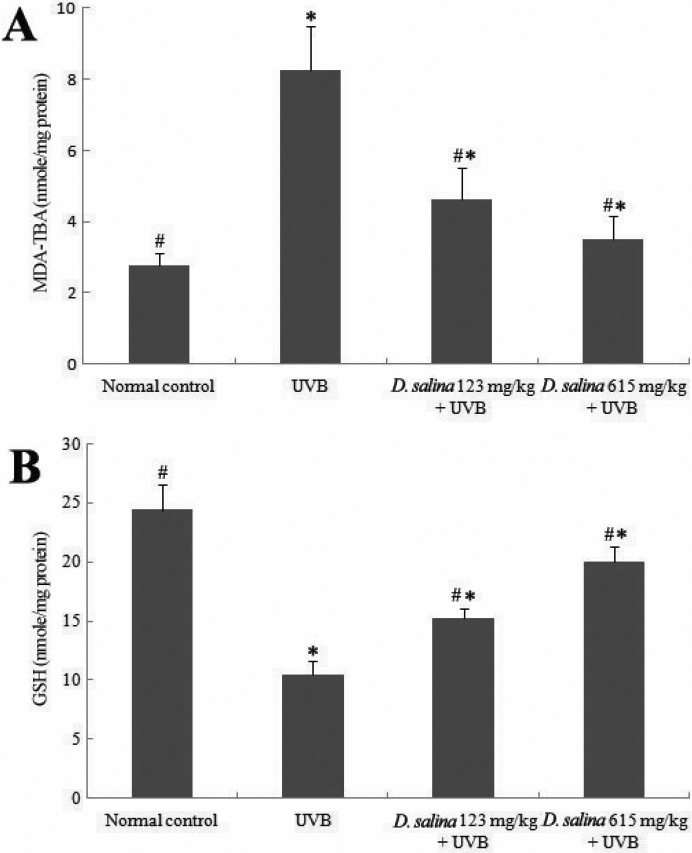
MDA-TBA and GSH levels. Effects of *D. salina* on corneal MDA-TBA (**A**) and GSH (**B**) levels in UVB-induced corneal oxidative damage in mice. Data are mean±SD of all the animals in a group; * p<0.05 compare with normal control; # p<0.05 compared with UVB-treated alone group.

MDA level is widely used as a marker of free radical-mediated lipid peroxidation injury. We measured MDA levels in the corneas, and the results are shown in Figure 4A. MDA levels in the UVB-treated group (8.27±1.24 nmole/mg protein) were significantly higher than those in the control group (2.78±0.32 nmole/mg protein, p<0.05). MDA levels in the *D. salina* treated group (4.62±0.88 and 3.52±0.64 nmole/mg protein at dose of 123 and 615 mg/kg, respectively) were significantly lower than those in the UVB-treated group (p<0.05).

GSH is an extremely efficient intracellular antioxidant against ROS that protects cells from UVB radiation damage. Therefore, the level of intracellular GSH is an important index for cellular antioxidative status. The results of the present study demonstrate that UVB irradiation caused a significant decrease in the GSH levels in the cornea (10.45±1.21 nmole/mg protein) as compared to the normal control group (24.38±2.14 nmole/mg protein). In contrast with the UVB-treated group, mice treated with *D. salina* at doses of 123 and 615 mg/kg showed significantly increased (46% and 91%) GSH levels (Figure 4B). These findings indicated that the free radicals being released in the cornea were effectively scavenged when treated with *D. salina.*

### Effect of *D. salina* on antioxidant enzyme activities after UVB exposure in the cornea

**Figure 5:**
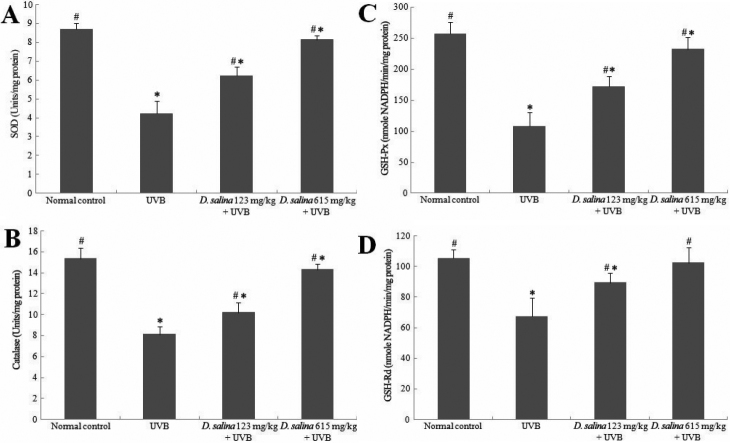
SOD, catalase, GSH-Px and GSH-Rd levels. Effect of *D. salina* on corneal antioxidant enzymes (**A**) SOD, (**B**) catalase, (**C**) GSH-Px, and (**D**) GSH-Rd in UVB-induced corneal oxidative damage in mice. Data are mean±SD of all the animals in a group; * p<0.05 compare with normal control; # p<0.05 compare with UVB-treated alone group.

To further elucidate the reduction of MDA accumulation in the UVB-exposed corneas, we examined the status of the antioxidant enzymes SOD, catalase, GSH-Px, and GSH-Rd in the corneas (Figure 5). The activities of corneal SOD, catalase, GSH-Px, and GSH-Rd in the UVB-treated group were significantly decreased by 51%, 47%, 58%, and 36%, respectively, when compared with the normal control group. In contrast, mice treated with 123 mg/kg of *D. salina* showed a significant increase in the activities of SOD, catalase, GSH-Px, and GSH-Rd by 47%, 26%, 59%, and 32%, respectively, as compared to the UVB-treated group. Similar results were also found in the dose of 615 mg/kg of *D. salina* (Figure 5). These data support a role for dietary *D. salina* in the depletion of MDA accumulation, through the increase of both antioxidant enzyme activities and GSH levels.

## Discussion

In our previous study, we observed that the major carotenoids of extract from *D. salina* were all-trans-β-carotene and 9- or 9’-cis-β-carotene, and the algal carotenoids extract had significantly higher antioxidant activity than all-trans forms of β-carotene, α-carotene, lutein, and zeaxanthin in all antioxidant assays [13]. We also reported that *D. salina* was effective in the prevention of CCl_4_-induced hepatic oxidative damage in mice, as evidenced by increased GSH amounts and antioxidant enzyme activity, such as SOD, catalase, and GSH-Px, and reduced MDA levels in the liver [7]. Therefore, we considered that *D. salina* may be useful in the prevention of various damages induced by oxidative stress. In the present study, the capability of *D. salina* to protect against UVB radiation-induced corneal damage and oxidative stress was investigated.

Earlier studies have demonstrated the harmful effects of UVB radiation from sunlight on the cornea [20,21]. UVB-induced reactive oxygen species, such as hydrogen peroxide, singlet oxygen, superoxide anions, and hydroxyl radicals, are reported to initiate peroxidation [22] and react to proteins or lipids, leading to membrane lipid peroxidation and, finally, cell necrosis [23,24]. The main ROS generation occurs in the cornea, due to high exposure to UVB radiation. Therefore, the corneal epithelium is the first line of UVB filtering capacity to absorb UVB radiation [25,26]. Several reports have indicated that an important mechanism of the protective effects in cornea may be related to the capacity of antioxidants to scavenge reactive oxygen species [14,27]. Indeed, a considerable body of literature has reported that several antioxidant agents, such as ascorbic acid [27] and zerumbone [28], reduce UVB-induced phototoxic effects in cornea by preventing oxidative stress. In the present study, we found that treatment with *D. salina* markedly inhibits UVB-induced corneal damage, as evidenced by cornea surface examination. The results of the cornea surface examination show that *D. salina* ameliorated UVB radiation-induced corneal damage.

The first protector of the optic axis is the cornea which, because it is rich in lipids, may lead to its more striking changes under oxidative stress. Lipid peroxidation by the generation of ROS is one of the principal mechanisms of UVB radiation-induced corneal injury [14]. Moreover, the initiation of oxidative stress related to various tissue injuries, cell death, and the progression of many acute and chronic diseases is generally believed to be induced by increased lipid peroxidation [29,30]. In experimentation, TBA reacts with MDA to form an adduct, a pink chromogen, which can be detected at 532 nm by spectrophotometer [31], and MDA is the major reactive aldehyde that appears during the peroxidation of biologic membrane polyunsaturated fatty acids [32]. An increase in MDA levels in the cornea suggests enhanced peroxidation, leading to tissue damage and failure of the antioxidant defense mechanisms to prevent the formation of excessive free radicals [30]. In the present study, UVB radiation-induced oxidative damage caused an increase in corneal MDA levels as compared to the normal control group. Treatment with *D. salina* significantly reversed these changes. The administration of *D. salina* caused a significant decrease in MDA levels compared to the UVB radiation-induced oxidative damage group.

Previous studies on the mechanism of UVB radiation-induced corneal damage showed that GSH acts as a nonenzymatic antioxidant that reduces H_2_O_2_, hydroperoxides (ROOH), and photooxidation [14]. GSH is easily oxidized to GSSG by xenobiotic compounds, and there may additionally be reaction with any of the selenium-containing GSH-Px isozymes, which may subsequently result in the reduction of GSH levels. GSSG is either rapidly reduced by GSH-Rd and NADPH or used in the protein-folding process in the endoplasmic reticulum. Because of these recycling mechanisms, GSH is an extremely efficient intracellular antioxidant for oxidative stress [33]. In the present study, the corneal content of GSH was significantly decreased in UVB radiation-exposed mice compared with control mice. Conversely, administration of *D. salina* to UVB radiation-exposed mice significantly elevated GSH content in the cornea compared to the untreated group, indicating that *D. salina* can protect against the UVB radiation-induced depletion of corneal GSH.

The balance of intracellular ROS depends on both their production within cells during normal aerobic metabolism and their removal by the antioxidant defense system that includes nonenzymatic antioxidants (e.g., GSH, bilirubin, and vitamins E and C) and enzymatic antioxidants such as SOD, catalase, GSH-Px, and GSH-Rd, in mammalian cells [14,34]. Therefore, the enzymatic antioxidant activities and/or the inhibition of free-radical generation are important in terms of protecting the cornea from UVB-induced oxidative damage [35]. Each of these enzymes catalyzes the reduction of a particular type of ROS. SOD is an exceedingly effective defense enzyme that catalyzes the dismutation of superoxide anions into hydrogen peroxide (H_2_O_2_) [36]. Catalase is a hemoprotein in all aerobic cells that metabolizes H_2_O_2_ into oxygen and water. GSH-Px is a selenoprotein that catalyzes the reduction of H_2_O_2_ and hydroperoxides to non-toxic products. GSH-Rd is a cytosolic hepatic enzyme involved in the detoxification of a range of xenobiotic compounds by their conjugation with GSH [7,37]. These antioxidant enzymes are easily deprived of their activity by lipid peroxides or free radicals, resulting in their decreased activities in UVB radiation exposure [35].

In the present study, SOD, catalase, GSH-Px, and GSH-Rd activities were significantly decreased in the cornea in response to UVB radiation treatment alone compared with normal control mice, implying increased oxidative damage to the cornea. In contrast, SOD, catalase, GSH-Px, and GSH-Rd levels were significantly elevated by administration of *D. salina* to UVB radiation-damaged corneas, suggesting that it has the ability to restore and/or maintain these enzymes’ activities in UVB radiation-damaged cornea.

Carotenoids, including hydrocarbons such as β-carotene and xanthophylls such as lutein and zeaxanthin, play an important role in protecting cells and organisms against the harmful effects of light, air, chemicals, and sensitizer pigments. The primary mechanism of action of this phenomenon appears to be the ability of carotenoids to quench excited sensitizer molecules and singlet oxygen [38]. Further, β-carotene protects liposomes against lipid autooxidation mediated by superoxide and hydroxyl radicals, as well as against lipid peroxidation and lysis caused by Fe^2+^-generated radicals (LO^●^ and LOO^●^) [39]. Recently, we demonstrated that *D. salina*, which contains abundant carotenoids and xanthophylls, is an efficient antioxidant against a variety of oxidative stress in vitro and in vivo [7,13]. Additionally, β-carotene is the major precursor of vitamin A, which protects corneal endothelial cells against iron-induced lipid peroxidation-mediated programmed cell death [40]. Under normal conditions, the conjunctival and scleral blood vessels in the limbus region are the major source of vitamin A for the cornea [41]. Therefore, carotenoids-enriched *D. salina* is expected to protect against UVB radiation-induced corneal oxidative damage.

In conclusion, the results of this study demonstrate that *D. salina* was effective in the prevention of UVB radiation-induced corneal oxidative stress in mice. Our results show that the protective effects of *D. salina* may be due to both an increase in the activity of the antioxidant defense system and an inhibition of lipid peroxidation. The inhibitory effects of dietary *D. salina* may be useful as a protective agent against UVB radiation-induced corneal damage in vivo.
